# Simple fabrication method for cancer cell migration studies on biomimetic substrates with tunable stiffness

**DOI:** 10.1016/j.mtbio.2026.103332

**Published:** 2026-06-08

**Authors:** Laura Sercia, Alberto Portone, Stefano Leporatti, Stefania Belli, Paola Franco, Maria Patrizia Stoppelli, Giuseppe Gigli, Alessandro Polini, Francesca Gervaso

**Affiliations:** a Institute of Nanotechnology, National Research Council (CNR-NANOTEC), C/o Campus Ecotekne, Via Monteroni, Lecce, 73100, Italy; b University of Salento, Dipartimento di Ingegneria Dell'Innovazione, C/o Campus Ecotekne, Via Monteroni, Lecce, 73100, Italy; c Tecnomed Puglia – Technopole for Precision Medicine (Biotech Lecce Hub), C/o Ecotekne Campus, Via Monteroni, Lecce, 73100, Italy; d Institute of Genetics and Biophysics “A. Buzzati Traverso” (CNR-IGB), National Research Council, Naples, 80131, Italy; e UniCamillus-Saint Camillus International University of Health Sciences, Departmental Faculty of Medicine and Surgery, Rome, 00131, Italy; f University of Salento, Dipartimento di Medicina Sperimentale, C/o Campus Ecotekne, Via Monteroni, Lecce, 73100, Italy

**Keywords:** Cell migration assay, In vitro models, Glioblastoma, Anti-migratory drugs, Hydrogel stiffness, Mechanobiology

## Abstract

Glioblastoma is one of the most invasive and aggressive brain cancers, yet current therapies fail to effectively target its pronounced migratory behavior. The development of anti-migratory strategies is therefore critical to improve patient outcomes, highlighting the urgent need for reliable in vitro platforms for drug testing. Here, a simple, low-cost, and reproducible hydrogel-based system that recapitulates key mechanical features of glioblastoma microenvironment is presented. By tuning hydrogel concentration, highly flat and transparent substrates with either uniform stiffness or controlled stiffness gradients are fabricated, enabling live-cell imaging and quantitative analysis of cell migration. Substrate stiffness modulates cell morphology, spreading, and motility in U87-MG glioblastoma cells and SVG-A astrocytes. To validate the platform for the screening of anti-migratory compounds, the cyclopeptide Ala-2/5, a recently developed anti-migratory molecule, is tested, revealing a significant reduction in cell migration in both cell types, with a stronger effect observed in tumor cells. Overall, these substrates overcome the limitations of traditional 2D assays while providing an easy-to-fabricate, versatile, and scalable tool for mechanobiology studies and for the preclinical evaluation of anti-migratory therapies targeting invasive cancers.

## Highlights

• Low-cost hydrogel platform mimics glioblastoma mechanics.
• Tunable uniform and gradient stiffness for migration assays.
• Flat, transparent substrates enable live-cell imaging.
• Ala-2/5 significantly reduces U87-MG cell migration.
• Scalable tool for anti-migratory drug screening.

## Graphical abstract

**Figure:**
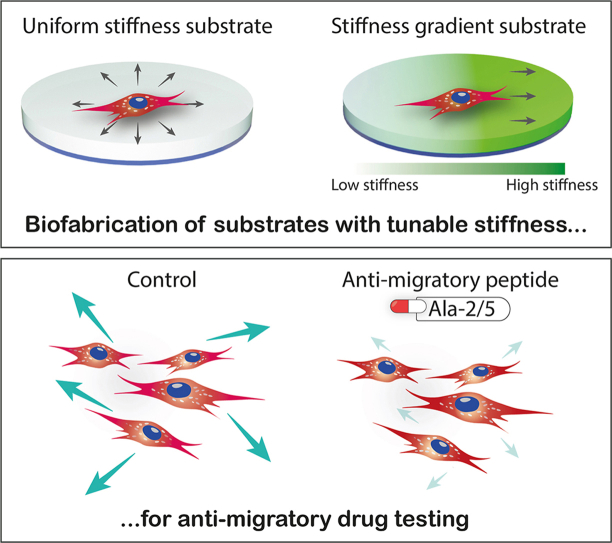

## 1 Introduction

Glioblastoma (GBM) is the most common and aggressive primary brain tumor in adults, accounting for approximately 15-20% of all brain malignancies [1,2]. It poses a major clinical challenge due to its rapid proliferation and highly invasive nature, often infiltrating surrounding brain tissue and crossing into the opposite hemisphere via the corpus callosum. This diffuse invasion makes complete surgical resection nearly impossible. Standard treatment includes maximal surgical debulking followed by radiation therapy and chemotherapy with temozolomide [3]. However, despite these aggressive multimodal strategies, GBM remains incurable, with a median survival of 12-15 months and a five-year survival rate below 5%. The high recurrence rate and poor prognosis are primarily driven by the tumor's ability to migrate and infiltrate normal brain tissue, allowing residual cells to evade treatment and repopulate the tumor [4,5].

The standard clinical treatments do not sufficiently target cell migration and invasion and there is an urgent need for effective anti-migratory therapeutic strategies. To develop and screen such treatments, there is a growing demand for in vitro models that recapitulate the physical and biochemical cues of the tumor microenvironment while remaining accessible, reproducible, and adaptable for drug testing. Traditional two-dimensional (2D) cell cultures fail to capture the complexity of in vivo tissue architecture and mechanical heterogeneity, often providing misleading results. In contrast, three-dimensional (3D) models based on biocompatible hydrogels, often structured by bioprinting [6] or included in organ-on-chip platforms [7], offer more physiologically relevant conditions by mimicking the extracellular matrix (ECM) and enabling the study of tumor cell behavior in a controlled environment enriched by specific physiological cues [[8], [9], [10]]. However, many of these systems are technically complex, require specialized equipment, or involve intricate fabrication protocols that hinder their widespread application in high-throughput studies.

In this context, we developed a simple, low-cost, and easily implementable in vitro system to investigate GBM cell migration using live-cell imaging. The fabrication process is rapid and allows for the realization of flat hydrogel substrates with uniform and controlled mechanical properties, suitable for real-time observation of cell motility by optical microscopy. Achieving a highly flat substrate is essential when studying cell migration driven specifically by stiffness cues, as it eliminates additional physical signals that could otherwise confound the results. Indeed, it has been widely demonstrated that even height variations as small as tens of nanometers can influence adhesion, cytoskeletal organization, and migration, thus, without a flat surface, cells may respond to topography rather than stiffness [11,12].

We used gelatin methacrylate (GelMA) hydrogel at different concentrations (from 5% to 20% w/v) to fabricate substrates for culturing U87-MG cells, a widely used GBM cell line, and SVG-A astrocytes. The range of GelMA concentration employed in the study fits with the typical stiffness of brain tumor tissue that reaches values of 13.5 kPa and up to 45 kPa during GBM tumor development [13,14], while spanning between 0.1 and 1 kPa in the healthy brain tissue [15].

GelMA, that is synthesized by grafting methacrylate groups onto the amine-containing side groups of gelatin, is widely used in brain cancer studies [[16], [17], [18], [19]] and offers several advantages in terms of stiffness modulation. Indeed, tuning the biopolymer concentration, the amount of methacrylate groups, and using a suitable photo-initiator, GelMA can be crosslinked through photocuring, creating a hydrogel with adjustable mechanical properties. Providing a culture environment that mimics mechanical properties of the physiological niche, we studied cell-substrate interaction on these flat substrates with uniform stiffness, evaluating cell morphology and spreading changes related to the substrate mechanical properties. Using dedicated image analysis tools, we evaluated also the cell motility, obtaining information on cell migration distance, velocity and direction. Moreover, leveraging the simplicity of the setup, we established a method to generate a stiffness gradient within the GelMA matrix, enabling the study of durotaxis, i.e. the directed migration of cells along stiffness gradients.

Finally, both (i) uniform-stiffness and (ii) gradient-based platforms were employed to assess the efficacy of a promising anti-migratory compound, the cyclopeptide [Ala2,Ala5]uPAcyclin (Ala-2/5) [20,21]. This molecule belongs to a family of peptides derived from a non-catalytic region of the human urokinase endowed with a regulatory function of cell migration [22]. One of these peptides, denoted uPAcyclin, reduced the number and size of lung metastases in a mouse model of tumor dissemination and is a strong inhibitor of pro-invasive activity of breast cancer-associated fibroblasts [23]. More recently, the [Ala2,Ala5]uPAcyclin potent derivative was developed and identified as an inhibitor of cell migration, matrix invasion and vasculogenic mimicry of GBM cells, using conventional migration/invasion assays (e.g., Boyden chamber assay) [21]. Thanks to our mechanically biomimetic platforms, we not only confirmed the peptide's anti-migratory activity but also gained deeper insight into its impact on cell migration, all through a simple and rapid in vitro assay.

## 2 Methods

### 2.1 Biofabrication of flat substrates with controlled stiffness

GelMA was synthetized from gelatin (type A, porcine skin, ∼175 bloom, Sigma Aldrich) following a previously established protocol [24], obtaining highly pure and lyophilized material. GelMA solutions at different concentrations (5%, 10%, 15%, 20% w/v) were obtained dissolving the biopolymer in a pre-heated phosphate-buffered saline (PBS), containing 10% w/v of FBS. The solutions were stirred at 50°C until complete dissolution, and then 0.5% w/v of LAP (Lithium phenyl-2,4,6-trimethylbenzoylphosphinate, Sigma Aldrich) was added. The resulting solutions were used for the fabrication of flat substrates with controlled stiffness. Briefly, a hydrogel drop (100 μL) was deposited on a flat polydimethylsiloxane (PDMS) cylindrical support produced using a 24-well plate well as a mold, cured at 50°C for at least 4 h, and washed three times with deionized (DI) water before use. To ensure uniform distribution, the drop was overlaid with a round glass coverslip (ø = 13 mm). The hydrogel was then exposed to UV light from the coverslip side, at 2.25 mW/cm^2^ for 60 s, to activate the LAP photoinitiator and induce crosslinking. The stiffness-gradient substrates were produced depositing two hydrogel drops, one composed of 10% w/v GelMA and the other of 20% w/v GelMA supplemented with 1 μg/mL of high molecular weight (70 kDa) dextran-FITC, on the flat PDMS support. The two drops were covered with a round glass coverslip (ø = 13 mm) and thermally treated at 50°C for 5 min before being exposed to UV light (2.25 mW/cm^2^ for 60 s). After UV crosslinking, the hydrogel surface in contact with the coverslip remained strongly attached to the substrate, whereas the side in contact with PDMS could be easily detached due to the hydrophobic nature of PDMS, obtaining a hydrogel slab adhered to the coverslip with a thickness of 1 mm. Therefore, the surface was used for subsequent characterization and cell culture.

To functionalize the surface and facilitate cell adhesion, only in case of culturing SVG-A cells, the substrates were coated with collagen type I from rat tail (Corning) with a final concentration of 10 μg/cm^2^.

To better assess the reproducibility of the fabrication process, we analyzed images from ten different devices produced at different times for independent experiments, acquired using various microscopy systems and imaging settings (e.g., automated tile scanning on a confocal microscope or imaging with a fluorescence stereo zoom microscope). The comparison of substrate photoluminescence (PL) intensity profiles along 10 mm lines spanning from the stiffer to the softer regions revealed a consistent gradient trend. For each position x, the mean PL intensity and standard deviation were calculated across the ten profiles. The coefficient of variation (CV = SD/mean) for the samples is reported as an inset in the graph.

### 2.2 Mechanical and topographical characterization

Two mechanical characterization methods were employed to evaluate substrates stiffness: (*i*) uniaxial unconfined stress–strain compression tests and (*ii*) atomic force microscopy (AFM) force/distance measurements.

For compressive tests, cylindrical structures (Ø ≃ 8 mm, h ≃ 5 mm) obtained from 5%, 10%, 15% and 20% GelMA solutions were produced using a PDMS mold (Fig. S1). The samples were hydrated in PBS or dH_2_O for 24h, then the diameter and thickness of the swollen cylinders were measured before starting the test. Each scaffold was tested with a Z5.0 testing machine (Zwick/Roell, Germany), equipped with a 10 N load cell. The swollen scaffolds were compressed at room temperature under displacement control up to 75% of strain with a rate of 0.02 mm s^−1^. A pre-load of 0.05 N was applied to ensure full contact between the sample and the testing surfaces and to define a clear and reproducible zero-displacement reference point. Following this, compressive stress–strain curves were recorded. At least 6 samples were tested for each GelMA concentration condition. The Young's modulus was calculated as the slope of the linear part of the stress–strain curve between 0% and 5% of the strain [25]. To better elucidate the mechanical behavior of less concentrated GelMA samples (GelMA 5% and 10% w/v), the analysis of the swelling ratio (SR) was also included. Swelling ratio (SR) was evaluated by determining the change in weight of freshly prepared and swollen hydrogels after 24 h of soaking in either PBS or dH_2_O at 37°C. Following separation from the PDMS mold, the initial weight *W*_*0*_ of the hydrogels was recorded. Hydrogels were subsequently placed in 12-well tissue culture plate and incubated in either PBS or dH_2_O at 37°C for up to 24 h. The weight of the swollen hydrogels *W*_*t*_ was recorded after 24 h by removing any excess liquid. The swelling ratio was calculated with the following formula:

$$Swelling\;ratio\;\left(SR\right)\left(\%\right)=\frac{W_{t}-W_{0}}{W_{0}}\times 100$$

The average diameters, measured by a caliper, of the swollen hydrogels after 24 h are reported for better clarity (Fig. S1d).

Force/distance and topographic surface roughness analysis were conducted by using a newly assembled Bio-Hybrid System called LEoPard (LEP), a combination of a JPK Nanowizard V Atomic Force Microscope (Bruker, Germany) coupled with a fluorescent and bright field microscope (Axio Observer, Zeiss, Germany), allocated inside of an environmental closed hood and positioned atop an anti-vibration table in order to minimize external noise [26,27]. AFM measurements were performed in liquid, at room temperature, by using a Scan Asyst-fluid cantilever (Bruker) with a triangular pyramid tip, nominal tip radius of 20 nm and spring constant of 0.7 N/m. The AFM scans were performed by using a soft-sample specific acquisition mode, namely “Quantitative imaging (QI™) advanced mode”, a force-spectroscopy-based imaging mode that collects maps of the sample, providing quantitative data on height, adhesion or mechanical properties. This AFM acquisition method records a complete force distance curve at each pixel of the image, indenting into the sample until a preset force is reached. We used a preset force of 5 nN and a z-length of 6 μm. Substrates with uniform stiffness were analyzed by collecting at least 3 maps (5 × 10 μm^2^, 30×60 pixels) for each sample. The Young's modulus of samples with controlled stiffness gradient was measured by acquiring maps in 13 positions, every 1 mm, along the direction of the gradient. To fit each Force vs Distance Curve pixel by pixel, by considering the radius of curvature of the indenter (e.g. AFM tip), a Hertz model was used as implemented in the JPK software. Topographic surface roughness (Rq, root mean square) analysis of the substrates was conducted in liquid and in tapping mode, using the same AFM setup and the same cantilever previously described, collecting randomly at least 3 separated images (5 × 10 μm^2^) per sample.

### 2.3 Cell culture

The U87-MG human glioblastoma and SVG-A human astrocyte cell lines were cultured in Dulbecco's Modified Eagle Medium (DMEM, Life Technologies) supplemented with 10% FBS (Life Technologies), in the presence of 100 U/mL of Na-penicillin and 100 μg/mL of streptomycin sulphate. The U87-MG cell line was purchased from ECACC (European Collection of Authenticated Cell Cultures, Salisbury, UK), whereas the SVG-A astrocyte cells were obtained from R.S. Lahue Laboratory, Human Molecular Genetics Lab, University of Galway, Ireland [28]. Cells were cultured at 37°C with 95% humidity and 5% CO_2_. After preliminary tests, a cell density of 5 × 10^5^ cells per cm^2^ and 6 × 10^6^ cells per cm^2^ were chosen for cell adhesion and cell migration studies respectively.

### 2.4 Generation of GFP-U87-MG and Tomato-SVG-A fluorescent cells

Stable transfection of 2.5 × 10^6^ subconfluent U87-MG cells was performed by electroporation with Neon™ Transfection System (Invitrogen) at 1300V, 30 msec, in the presence of 30 μg of pEGFP-N1 vector (Real Gene Biotech Company), encoding green fluorescent protein (GFP) in 3 mL DMEM. After transfection, U87-MG cells were seeded in DMEM/10% FBS without antibiotics for 6 h and then in DMEM/10% FBS with antibiotics. In contrast, SVG-A astrocytes were transfected with a Tomato fluorescent reporter plasmid (ptdTomato-N1, Clontech) using the Transfectin® Lipid Reagent (Bio-Rad, Hercules, CA, USA), according to the manufacturer's instructions. Briefly, plasmid DNA and Transfectin® reagent were separately diluted in serum-free DMEM, combined, and incubated for 20 min at room temperature to allow complex formation. This mixture was then added dropwise to 60–70% confluent SVG-A cells in 100 mm plates and, after 4-6 h, the medium was replaced with complete DMEM/10% FBS.

Two days post-transfection, both cell lines received the neomycin analogue G418 (Sigma) at a concentration of 0.8 mg/mL, twice a week, for three weeks to select for a pool of G418-resistant cells. Evaluation of the transfection efficiency, as a percentage of fluorescent cells over total cell number indicated that approximately 70% of U87-MG and 30% of SVG-A cells were successfully transfected.

### 2.5 Immunofluorescence staining

Immunofluorescence analysis was performed on cells cultured onto uniform GelMA substrates (5%, 10%, 15%, 20% w/v) or a standard polystyrene cell culture substrate (as a control) for 72 h at 37°C with 5% CO_2_. Briefly, the cell-culture substrates were washed twice with PBS, fixed with 4% PFA, and permeabilized in 0.2% PBS/Triton X-100 for 15 min. Blocking solution (2% BSA in 0.1% PBS/Triton) was added for 1 h at room temperature. U87-MG and SVG-A cells were stained with FITC-Phalloidin for 25 min at room temperature. Cell nuclei were stained with DAPI. Fluorescent images were obtained by a Carl Zeiss confocal microscope (LSM700) using EC Plan-Neofluar 10×/0.45 objective and 40×/1.3 oil immersion objective. Fluorescent images were further processed and analyzed using ImageJ (version 1.54p) to quantify different morphological parameters including the nuclear and cell area, nuclear aspect ratio, and circularity.

### 2.6 Live imaging and migration analysis

U87-MG and SVG-A control or treated cells (exposed to 100 nM Ala-2/5) were seeded on GelMA substrates (10%, 20%, gradient), let adhere for 24 h, and then monitored by confocal time-lapse imaging (Zeiss LSM980 confocal microscope) using a Clr Plan-Apochromat 10×/0.5 objective. Cells were maintained at 37°C with 5% CO_2_ over a total period of 14 h using the integrated incubation modules for long-term live cell imaging with stable temperature conditions, capturing images every 30 min. For both uniform and stiffness gradient substrates, a minimum of three fields per sample were imaged. For the stiffness gradient substrates specifically, the field were randomly selected along the gradient. Reported results derive from at least three independent experiments. Tracking analysis was performed using the automated tracking software TrackMate 7 distributed as a Fiji plugin [18]. To ensure reliable results, migration analysis was limited to cells that were continuously tracked for 8 h, thereby excluding short tracks caused by out-of-focus cells or debris. This approach ensured a robust dataset, with approximately 150 valid cell tracks analyzed per sample. The obtained migration tracks were plotted using chemotaxis and migration tool distributed as a Fiji plugin (Ibidi GmbH, Martinsried, Germany, version 1.01). Several values characterizing cell migration were computed from the trajectories by the software, such as accumulated distance (*i.e.* the total cell path), velocity, and movement angle direction. The velocity was calculated as the ratio of the accumulated distance of the cell path, and the time of migration. Data were visualized with GraphPad Prism v.10. Accumulated distance and tracks velocity data are displayed as Violin plots, while movement angle directions are displayed as Rose plots.

### 2.7 Quantitative analysis of filopodia length and orientation

Individual filopodia were identified from brightfield images of U87-MG and SVG-A control or treated cells (100 nM Ala-2/5) cultured on GelMA substrates (10%, 20%, and gradient), acquired by confocal time-lapse imaging. At least 10 frames of 3 different sample areas have been used for the analysis. Filopodia were manually delineated to quantify their length, number, and orientation. The analysis was performed across three independent experiments, with approximately 300 filopodia measured per condition. The proportion of filopodia per cell was calculated by normalizing the total number of filopodia to the number of cells within the analyzed areas. Filopodia length and number are presented as bar plots. Elongation angles were determined by measuring the orientation of the traced filopodia using Fiji and are displayed as rose plots.

### 2.8 Statistical analyses

All the experiments were performed in at least three independent replicates, and data are presented as mean ± standard error (SEM) or ± standard deviation (SD), as reported specifically in each figure legends. GraphPad Prism v.10 was used to test statistical differences with one-way, or two-way ANOVA tests, considering p = 0.05 as the level of significance. Significantly different results are indicated in the graphs with stars (∗p < 0.05; ∗∗p < 0.01; ∗∗∗p < 0.001; ∗∗∗∗p < 0.0001).

## 3 Results

### 3.1 Simple and fast method for the fabrication of flat GelMA substrates with different stiffness

**Fig. 1:**
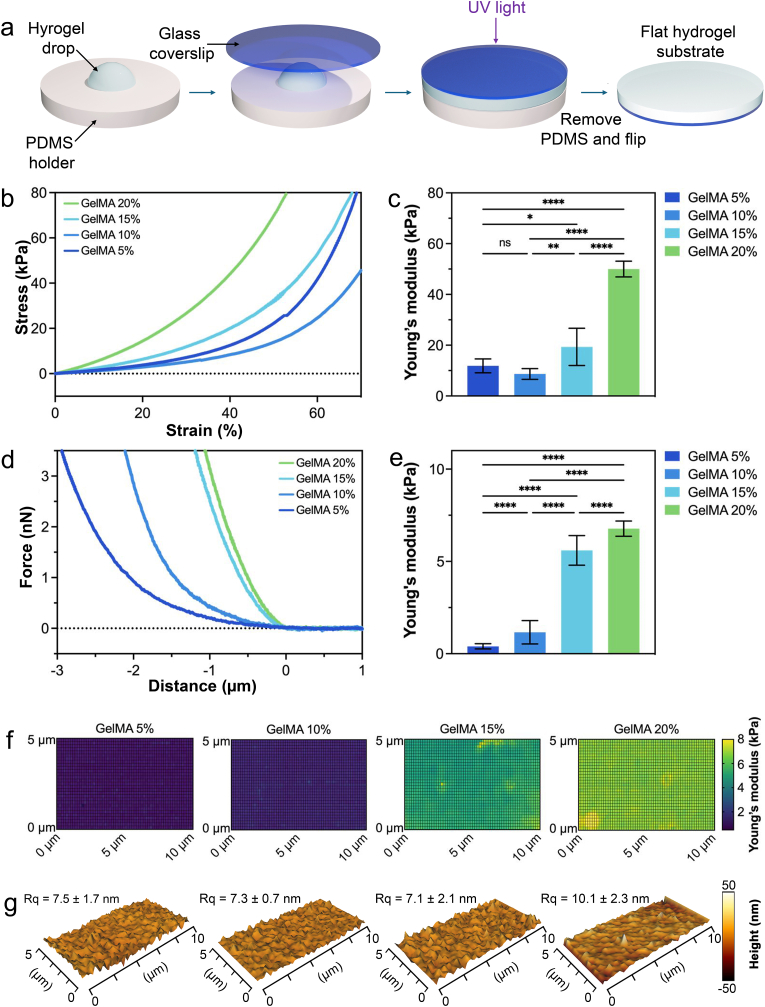
a) Schematic representation of GelMA substrates fabrication process. b) Mechanical properties characterization performed by compression tests (b,c) and force/distance AFM (d,e). Graphs show examples of the stress/strain (b) and force/distance (d) curves obtained during the measurements and the relative Young's Moduli calculated from the curves (c, e). f) Stiffness maps collected through force/distance measurements in QI mode AFM (5 × 10 μm^2^, 30×60 pixels), with every pixel corresponding to a force/distance measurement. g) Topographic surface roughness (Rq, root-mean-square) of the substrates evaluated by tapping mode AFM (5 × 10 μm^2^ images in a three dimensional-like view). Values denote mean ± SEM. ∗p < 0.05; ∗∗p < 0.01; ∗∗∗∗p < 0.0001; ns = not significant.

To model the biomechanical properties of tumor microenvironment in an in vitro model, we developed a simple strategy to fabricate GelMA substrates with different stiffness in the range of physiological or tumorigenic brain tissues. As shown in Fig. 1a, a flat hydrogel substrate was easily obtained by depositing a hydrogel drop of the desired concentration (5%, 10%, 15%, 20% w/v) on a flat PDMS support, covering with a glass coverslip, and later exposed to UV light. Substrates stiffness was evaluated through compressive mechanical tests, obtaining the force/deformation curves reported in Fig. 1b and the relatives Young's Modulus reported in Fig. 1c. The graphs show a clear increase in stiffness from 10% to 20% w/v GelMA, from approximately 10 to 50 kPa (8.7 ± 1.9 kPa; 19.4 ± 6.6 kPa; 50.0 ± 2.7 kPa, for 10%, 15% and 20% GelMA samples, respectively), in agreement with previously reported data [29]. In contrast, the 5% w/v sample exhibits a stiffness of 11.9 ± 2.6 kPa, which is unexpectedly similar to that of the 10% sample. Such deviation from the expected trend was attributed to specific arrangement of GelMA polymer chains, observable at low concentrations and in the presence of saline solutions such as PBS [30,31]. Indeed, to ensure that the measured mechanical properties reflect conditions close to those of practical use, all measurements were performed after 24 h of immersion in PBS. Furthermore GelMA 5% and 10% samples were also analyzed after immersion in either PBS or dH_2_O at 37°C for up to 24 h. GelMA 5% sample were found to swell significantly less in PBS than in water (Fig. S1) (SR_*PBS*_ = −35.4 ± 1.4% and SR_*H2O*_ = −10.2 ± 2.2%). This behavior could be attributed to the different ionic strength of the surrounding media, which strongly affects the swelling of gelatin-based hydrogels. In PBS, the presence of salts screens electrostatic repulsions along the polymer chains and promotes a more compact network conformation, resulting in reduced swelling and a higher effective crosslinking density. This translates into improved mechanical properties, as reflected by the higher Young's modulus measured in PBS compared to dH_2_O (E_low_ _*PBS*_ = 11.9 ± 1.1 kPa and E_low__*H2O*_ = 7.0 ± 0.6 kPa). Conversely, in pure water, the lack of ionic screening leads to increased chain expansion and swelling, which lowers the network density and consequently reduces the stiffness of the hydrogel. No significant differences were found for GelMA 10% samples, which swelling behavior and mechanical properties were similar after immersion in either PBS or dH_2_O. Here, the higher polymer concentration leads to an increased crosslinking density and a more constrained network structure. As a result, the hydrogel exhibits a reduced sensitivity to the ionic strength of the surrounding medium, limiting chain mobility and preventing significant differences in swelling between PBS and water. Consequently, the mechanical properties remain largely unaffected, as the already dense network minimizes further conformational changes induced by the external environment.

Notably, this behavior was not observed in the force/distance measurements obtained by AFM (Fig. 1d and e), which instead show a clear and consistent increase in Young's modulus, related to increasing GelMA concentration (0.4 ± 0.1; 1.2 ± 0.2; 5.6 ± 0.8; 6.8 ± 0.4 kPa, from 5% to 20% GelMA). High-resolution force/distance maps acquired in AFM-QI mode (Fig. 1f) demonstrated the high uniformity of substrate stiffness across the sample surface. AFM analysis also allowed the assessment of substrate roughness, revealing remarkably smooth surfaces with Rq values of 7.5 ± 1.7 nm, 7.3 ± 0.7 nm, 7.1 ± 2.1 nm, and 10.1 ± 2.3 nm for the 5%, 10%, 15%, and 20% w/v substrates, respectively. For comparison, we measured surface roughness of cell culture Petri dishes using the same protocol and parameters, obtaining similar values (Rq = 6.3 ± 0.5 nm).

### 3.2 Matrix stiffness strongly influences cell shape and adhesion

**Fig. 2:**
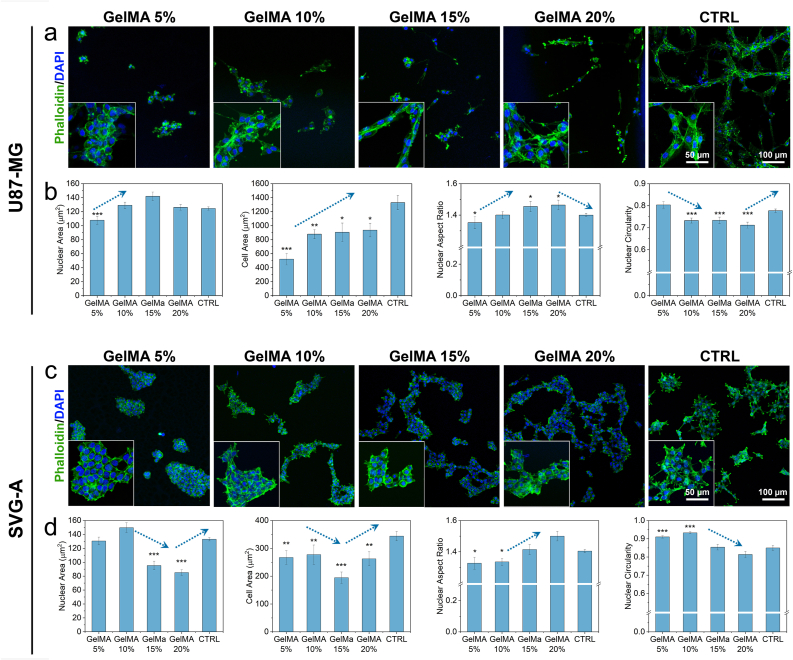
U87-MG (a,b) and SVG-A (c,d) confocal micrographs (a,c) and morphological analysis (b,d). Cells were cultured on GelMA substrates with increasing stiffness (from 5% to 20% w/v) for 72 h. Cells grown on plastic plates were used as a control (CTRL). Cell cytoskeleton is marked in green and nuclei in blue. Scale bar = 100 μm. Insets show higher magnification views of the same sample. Scale bar = 50 μm. Pictures are representative of what was observed in at least three independent experiments. Graphs (b,d) reporting nuclear and cell area, nuclear aspect ratio and circularity are based on measurements from more than 100 cells, using at least 10 images per sample (n ≥ 10). Trend variations of morphological indicators (b,d) are highlighted by blue dashed arrows. Values denote mean ± SEM. ∗p < 0.05; ∗∗p < 0.01; ∗∗∗p < 0.001.

U87-MG and SVG-A cells were cultured on GelMA substrates (5%, 10%, 15%, and 20% w/v) with different stiffness to investigate the effect of the mechanical stimulus on cell shape and adhesion. To this end, we performed fluorescent staining using DAPI and phalloidin and quantified several morphological parameters including nuclear and cell area, nuclear aspect ratio, and circularity (Fig. 2). Compared to the control condition, represented by a standard polystyrene cell culture substrate [[32], [33], [34]], U87-MG cells grown on 5% w/v GelMA exhibit a significantly smaller nuclear area (124.5 ± 2.4 μm^2^ and 107.6 ± 5.6 μm^2^, respectively). From 10% w/v to 20% w/v GelMA, nuclear size assumes values comparable to the control (129.1 ± 4.4, 141.9 ± 6.2, 126.1 ± 4.0 μm^2^ for GelMA 10%, 15%, and 20% w/v, respectively).

Changes in nuclear area are generally accompanied by corresponding variations in overall cell area. Indeed, analysis of the total area of individual cells reveals a marked reduction on 5% GelMA substrates (521.0 ± 82.5 μm^2^) compared to the control (1329.6 ± 99.9 μm^2^). Substrates with 10%, 15%, and 20% w/v GelMA show intermediate values (878.5 ± 63.2 μm^2^, 904.9 ± 131.9 μm^2^, 935.2 ± 96.8 μm^2^, respectively) with a clear upward trend related to GelMA concentration. Interestingly, the nuclear aspect ratio, a parameter indicative of nuclear elongation and commonly reflective of overall cell elongation, increases progressively from 5% (1.35 ± 0.04) to 15% (1.45 ± 0.03), it is similar between 15% and 20% (1.46 ± 0.03), and then significantly decreases in the control (1.40 ± 0.01). As expected, this trend is inverted in the analysis of nuclear circularity, which reveals more circular nuclei on the 5% substrates, a shift toward more elliptical shapes from 10% to 20% samples, and a return to more circular nuclei in the control condition.

The same analysis was performed on SVG-A cells (Fig. 2d) cultured on collagen-coated GelMA substrates (5%, 10%, 15%, and 20% w/v) to allow cell adhesion. To assess eventual coating-related variations in substrate stiffness and topography, we evaluated these features by AFM measurements (Fig. S2). No significant differences have been detected between coated and non-coated substrates. This is likely due to the limited thickness of the collagen layer deposited on the surface. While this layer is sufficient to promote SVG-A cell adhesion, it is not sufficient to significantly alter the overall surface mechanical properties and morphology. The collected data show a significantly smaller nuclear area when cells are cultured on GelMA 15% and 20% (95.2 ± 4.2 μm^2^, 85.2 ± 2.2 μm^2^) correlated with a clear reduction of the total cell area (194.6 ± 20.3 μm^2^, 262.3 ± 25.3 μm^2^) compared to the control (nuclear area: 133.0 ± 6.8 μm^2^, cell area: 343.8 ± 16.9 μm^2^).

Substrates with 5% and 10% GelMA show small values of nuclear aspect ratio (1.33 ± 0.03, 1.33 ± 0.04) but starting from GelMA 15% the nuclear aspect ratio approaches values comparable to the control (1.40 ± 0.06). Conversely, the nuclear circularity is higher for the cells grown on softest substrates (0.91 ± 0.01 μm^2^, 0.93 ± 0.01 μm^2^, for GelMA 5% and 10%, respectively) and decreases in GelMA 15% and 20% w/v (0.85 ± 0.01 and 0.81 ± 0.02), reaching the value observed in control samples (0.85 ± 0.01).

These morphological parameters provide a clear and quantitative description of cell adhesion across the different substrates. Indeed, both U87-MG and SVG-A cells on the softest substrates (5% GelMA) appear more rounded, with limited spreading. As GelMA concentration increases, cells spread more extensively on the substrate and adopt an anisotropically elongated morphology, reflected by increased area and aspect ratio, and reduced circularity. On the control polystyrene substrate, however, we observed a significant increase in cell area, but with a more isotropic spreading, which is also mirrored in the nuclear morphology, showing a more rounded and less elongated shape.

The morphology of SVG-A cells on 5% substrates is strongly influenced by their growth in clusters. On these substrates, the clusters exhibit rounded borders, large nuclear areas, and cells appear more strongly bound to each other than to the substrate. As substrate stiffness increases, cell adhesion to the substrate becomes more pronounced. This is accompanied by a reduction in both nuclear and cell area, as well as an increase in aspect ratio and cell elongation, reflecting enhanced cell spreading.

### 3.3 Cell migration is enhanced on stiffer substrates

**Fig. 3:**
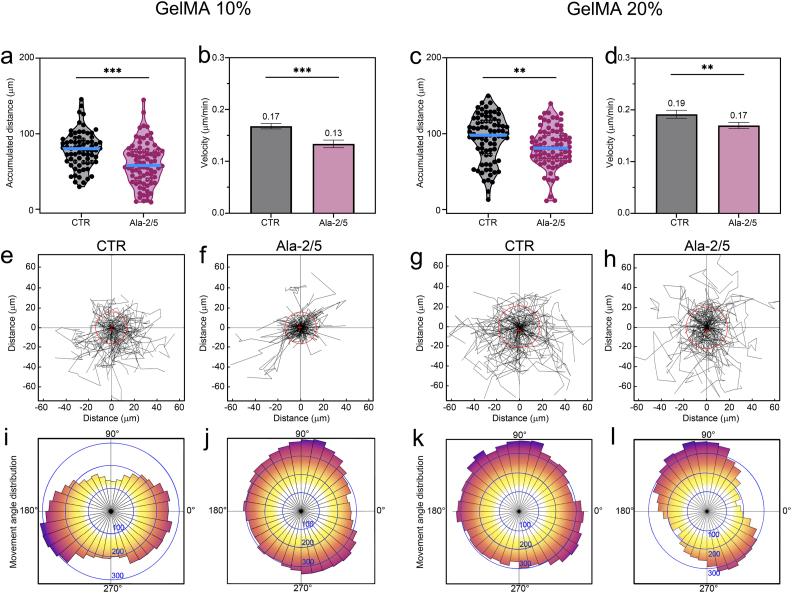
Quantitative analysis of U87-MG cell migration on 10% and 20% GelMA substrates with or without Ala-2/5 treatment. a-d) Histograms reporting the accumulated distance (a,c) and migration velocity (b,d). e-h) Cell migration trajectories. Red circles are visual guides to emphasize the differences among the samples. i-l) Rose plots showing the angular distribution of all movements within the analyzed tracks. Blue circles indicate the frequency of movements associated with each angle. Over 150 cell tracks were analyzed per sample. The analysis was obtained from at least three independent experiments. Values denote mean ± SEM. ∗p < 0.05; ∗∗p < 0.01; ∗∗∗p < 0.001.

Given the lower adhesion capacity of cells plated on softer substrates (GelMA 5%), cell migration was assessed only on GelMA 10% and GelMA 20% w/v samples, whose stiffness values broadly cover the range reported for GBM tumor tissue [13,14]. Thus, U87-MG cells were seeded on the selected substrates, allowed to adhere for 24 h, and then monitored using live-cell imaging for a total of 14 h. Using a dedicated image analysis tool (see materials and methods section), we quantified cell motility, migration distance, velocity, and direction in both control and treated samples. We also employed the platform to test the effects of the anti-migratory peptide uPAcyclin-II (Ala-2/5) [20,21] on U87-MG glioblastoma cells, aiming to assess changes in the migratory behavior (Fig. 3). Interestingly, the accumulated distance of GBM cells is lower when seeded on 10% GelMA (79.1 ± 2.8 μm) in comparison to 20% GelMA (93.5 ± 3.5 μm). As a result, also the measured velocity shows the same trend. Moreover, when the cells are exposed to the anti-migratory peptide (Ala-2/5), the migration is impaired: indeed, the accumulated distance and the velocity significantly decrease both in the soft (10% w/v; distance = 60.1 ± 3.1 μm) and in the stiff (20% w/v; distance = 81.6 ± 2.6 μm) GelMA substrates (Fig. 3a–d). Moreover, the reported cell trajectory graphs (Fig. 3e–h) show a clear reduction of migration when U87-MG are treated with Ala-2/5 especially in 10% GelMA substrates. Finally, the analysis of movement angle distribution, reported in the rose plots of Fig. 3i–l, demonstrates a random migration in both the control and the treated samples.

**Fig. 4:**
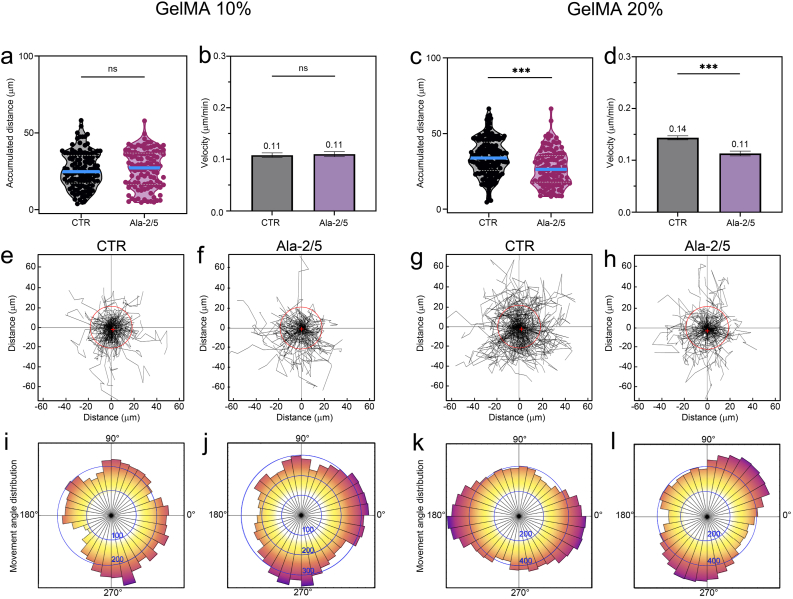
Quantitative analysis of SVG-A cell migration on 10% and 20% GelMA substrates with or without Ala-2/5 treatment. a-d) Histograms reporting the accumulated distance (a,c) and migration velocity (b,d). e-h) Cell migration trajectories. Red circles are visual guides to emphasize the differences among the samples. i-l) Rose plots showing the angular distribution of all movements within the analyzed tracks. Blue circles indicate the frequency of movements associated with each angle. Over 150 cell tracks were analyzed per sample. The analysis is obtained from at least three independent experiments. Values denote mean ± SEM. ∗p < 0.05; ∗∗p < 0.01; ∗∗∗p < 0.001; ns = not significant.

The same characterizations were performed using a human immortalized astrocytic cell line (SVG-A) to analyze the migration of non-tumoral glial cells derived from brain. As reported in Fig. 4 the anti-migratory peptide does not affect migration of SVG-A cells when they are cultured on 10% GelMA substrates (Fig. 4a and b). Conversely, a slight reduction of accumulated distance (Fig. 4c) and velocity (Fig. 4d) is detectable when cells are seeded on 20% samples. As previously observed for U87-MG cells, the direction of migration is isotropic; no preferential migration angle is detected in the analyzed samples, likely due to the homogeneous stiffness of the substrates (Fig. 4i–l).

To evaluate whether cell proliferation influences the migration data, we quantified changes in cell number over the course of the analysis by counting the number of cells within the field of view at different time points. The results (Fig. S3) showed that cell number fluctuates within approximately ±15% relative to the initial value for both cell types and both substrate types (10% and 20% GelMA). This variation is likely attributable to cell movement; indeed no clear growth trend was observed, indicating that the acquisition time is too short to capture measurable proliferation effects. Consequently, the contribution of proliferation to migration analysis can be considered negligible.

### 3.4 Simple and rapid fabrication of substrates with a stiffness gradient

**Fig. 5:**
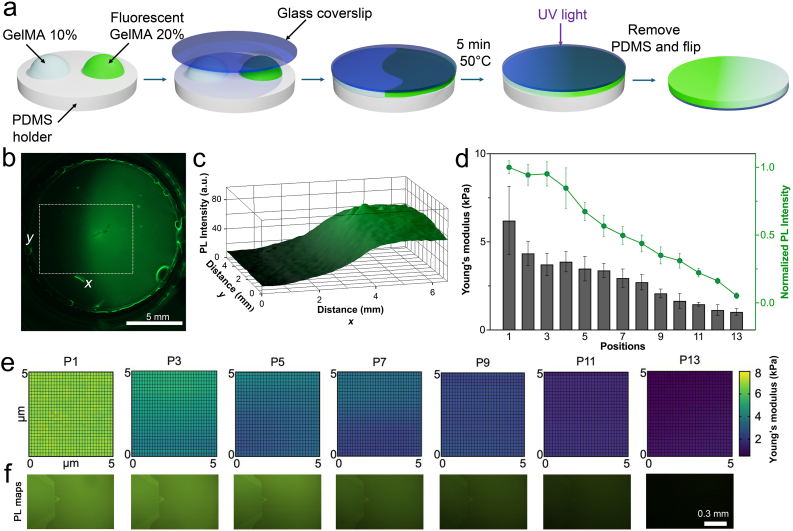
Schematic representation of the fabrication process employed for fabricating the stiffness gradient GelMA substrates (a). Gradient analysis performed by fluorescent microscopy using high molecular weight dextran-FITC labeled-20% w/v GelMA (b), PL intensity map (c) of the area under the dashed square in b, and AFM mechanical characterization (d). e) Stiffness maps collected through force/distance measurements in QI mode AFM (5 × 5 μm^2^, 30×30 pixels); every pixel corresponds to a force/distance measurement. f) Fluorescent micrographs of the relative area (P1-P13) acquired using a 488 nm filter on the AFM microscope. Values denote mean ± SEM. Scale bar 0.3 mm.

Migration in vitro models typically provide a driving force, such as a chemotactic [35,36] or a stiffness gradient, to direct cell migration. We implemented our method, maintaining a simple fabrication strategy while introducing a linear stiffness gradient to act as a mechanical driving force, guiding cell movement along a preferential direction. The graphical scheme of Fig. 5a reports on the fabrication process. After deposition of two drops of 10% and 20% w/v GelMA on the PDMS support, a glass coverslip is placed on the drops. Then a fast thermal treatment at 50°C for 5 min, prior to UV crosslinking, guarantees a partial mixing between the two hydrogel formulations, resulting in a substrate with a stiffness gradient. The addition of high molecular weight dextran-FITC at low concentration to the 20% GelMA allows to mark the stiffer component and visualize the gradient formation by fluorescent microscopy (Fig. 5b). Fig. 5c shows the photoluminescence (PL) intensity map of the area under the dashed square in Fig. 5b and clearly demonstrates the presence of a gradient of PL emission in the central region of the sample. To confirm the correlation of the PL gradient with the substrate stiffness, the samples were characterized by AFM in force/distance mode. The analysis confirms the formation of the stiffness gradient, with a Young's modulus spanning approximately from 1 to 6.5 kPa and demonstrates also a good correlation between fluorescence intensity (green dots in Fig. 5d) and the local Young's modulus (gray bars in Fig. 5d) trends. High-resolution force/distance maps, acquired in AFM-QI mode (Fig. 5e), show a clear and consistent gradient in Young's modulus across the entire sample, from the first position (P1, in Fig. 5e) to the last one acquired (P13, in Fig. 5e). The stiffness gradient is unambiguously associated with a decrease of the fluorescence emission of dextran-FITC-labeled GelMA 20% w/v, across the entire sample, from the first position (P1, in Fig. 5f) to the last one acquired (P13, in Fig. 5f), demonstrating the correlation between the PL gradient with the substrate stiffness.

To mathematically correlate PL intensity with stiffness, we performed a linear fit of the normalized fluorescence profile (PL) and of the Young's modulus (E), measured by AFM force–distance curves and expressed in kPa. The following relationships were obtained:

(1) PL=−0.078x+1.09

(2) E=−322.015x+5.054

By combining these equations, we derived a direct correlation between normalized PL intensity and Young's modulus:

E=4.1PL+0.554

To better assess the reproducibility of the fabrication process proposed, we analyzed images from ten different devices fabricated at different times and for different experiments, comparing their photoluminescence (PL) intensity profiles along 10 mm lines spanning from the stiffer to the softer regions (Fig. S4). PL intensity mean and standard deviation were also determined across the ten profiles giving a coefficient of variation of 11.2%, indicating good reproducibility of the fabrication process.

**Fig. 6:**
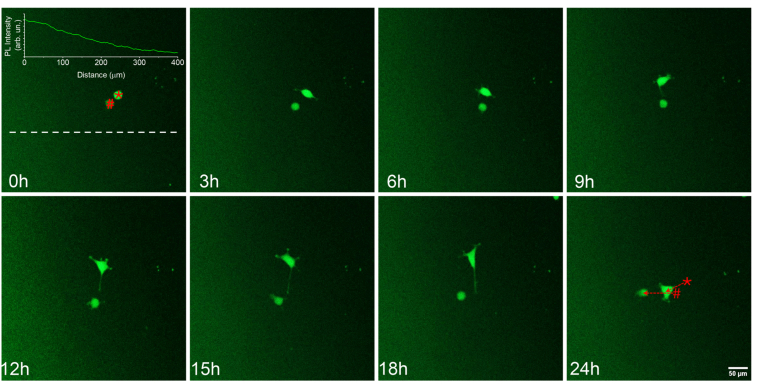
Exemplary micrographs, acquired at different timepoints, of GFP labeled-U87-MG moving on a graded substrate. The green background with increasing intensity from right to left, is due to the presence of dextran-FITC in GelMA 20% solution and marks the stiffness gradient that grows with PL intensity. The inset in 0 h frame shows the PL intensity profile acquired along the dashed line. Red arrows in the 24 h frame show the Euclidean distance traveled by cells. ∗ and # indicate the starting position of cells in t0 frame.

Both U87-MG and SVG-A cells were seeded on graded substrates and imaged by live imaging to study the influence of stiffness gradient on cell migration. The panel in Fig. 6 reports a representative example of the conducted analysis. The live imaging acquisition shows two GFP-labeled U87-MG cells at different time points. The green fluorescence of the background increases from right to left, as demonstrated by PL intensity profile plotted in the inset of t0 frame. Cells moved in an apparently random manner, producing filopodia and exploring their surroundings. Comparing the position of the cells at time 0h (indicated by ∗ and #) with their final position after 24 h, overall, they moved towards the stiffer region of the substrate (red arrows in 24 h frame in Fig. 6).

**Fig. 7:**
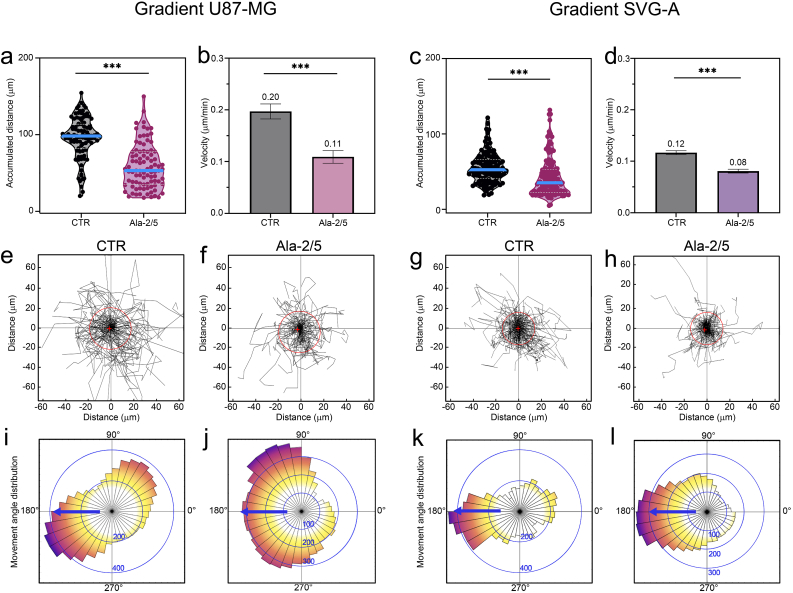
Quantitative analysis of U87-MG and SVG-A cell migration on GelMA stiffness-gradient substrates, with or without Ala-2/5 treatment. a-d) Histograms reporting the accumulated distance (a,c) and migration velocity (b,d). (e-h) Cell migration trajectories. Red circles are visual guides to emphasize the differences among the samples. (i-l) Rose plots showing the angular distribution of all movements within the analyzed tracks. Blue circles indicate the frequency of movements associated with each angle. Over 150 cell tracks were analyzed per sample. n = 3, the analysis was obtained from at least three independent experiments. For better clarity, stiffer GelMA substrates (20% w/v) were arbitrarily positioned on the left side for each sample, as indicated by the blue arrows. Values denote mean ± SEM. ∗p < 0.05; ∗∗p < 0.01; ∗∗∗p < 0.001.

The migratory behavior of U87-MG and SVG-A cells seeded on graded substrates, either treated or not treated with the anti-migratory peptide, was thoroughly characterized, and the results are shown in Fig. 7. The analysis demonstrates that Ala-2/5 reduces both the distance traveled by the cells and their migration velocity. This effect is more pronounced in U87-MG cancer cells than in SVG-A cells (Fig. 7a–d) and is further supported by cell trajectory plots (Fig. 7e–h) in which is clearly visible that treated cells exhibit reduced migration, covering a shorter distance and a more confined area compared with non-treated samples. Interestingly, the migration direction of both cell types is strongly influenced by the substrate stiffness gradient. The analysis of the movement angle distribution reveals that most cell displacements occur from the softer region (right side of the wind-rose plots, Fig. 7i–l) toward the stiffer region (left side). This directional anisotropy appears more evident in SVG-A cells than in U87-MG cells, providing insights not only into the drug's anti-migratory effect, but also into the cell sensitivity to substrate stiffness.

These findings derive from the overall migratory behavior detected in different regions of the gradient. However, the developed imaging and analysis strategy also allows to evaluate cell migration in a specific region of the gradient, correlating the migration to a specific range of stiffness of the single analyzed field. To demonstrate this additional potentiality of the proposed platform, we investigated U87-MG migration in 5 regions across the gradient. Results are reported in Fig. S5 and show that the average cellular behavior is consistent with the trends reported in Fig. 3, Fig. 4, Fig. 7. The difference of stiffness in every region has been evaluated using the formula in Equation (3) and correlating the fluorescence emission profile to the Young's modulus. More in detail, cells located in stiffer regions exhibit increased migration speed and distance, whereas cells in softer regions show reduced motility (Fig. S5a–c). Also, directionality of migration seems more pronounced on stiffer regions.

**Fig. 8:**
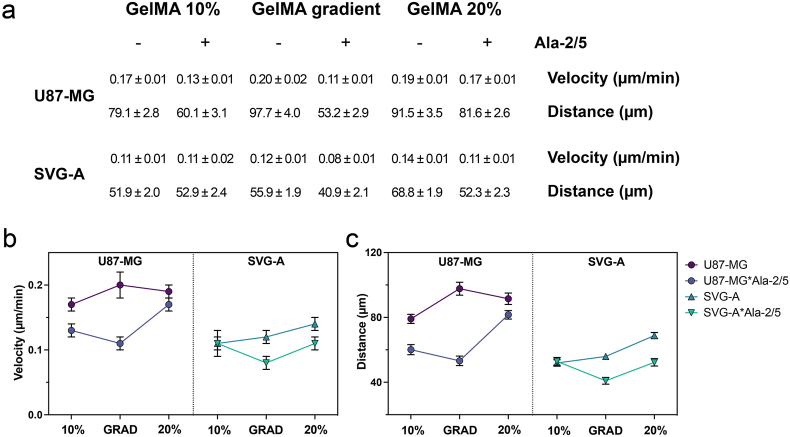
Measured cell velocities and accumulated migration distances for U87-MG and SVG-A cells cultured on the different substrates, with or without Ala-2/5 treatment, are summarized in the table (a), and in the velocity and distance graphs (b,c). Values denote mean ± SEM.

Fig. 8 reports the overall results for easier comparison. In summary, across all tested substrates, the drug treatment is able to reduce both migration velocity and distance in U87-MG cells, whereas in SVG-A cells this effect is observed only on stiffer (GelMA 20%) or gradient substrates.

**Fig. 9:**
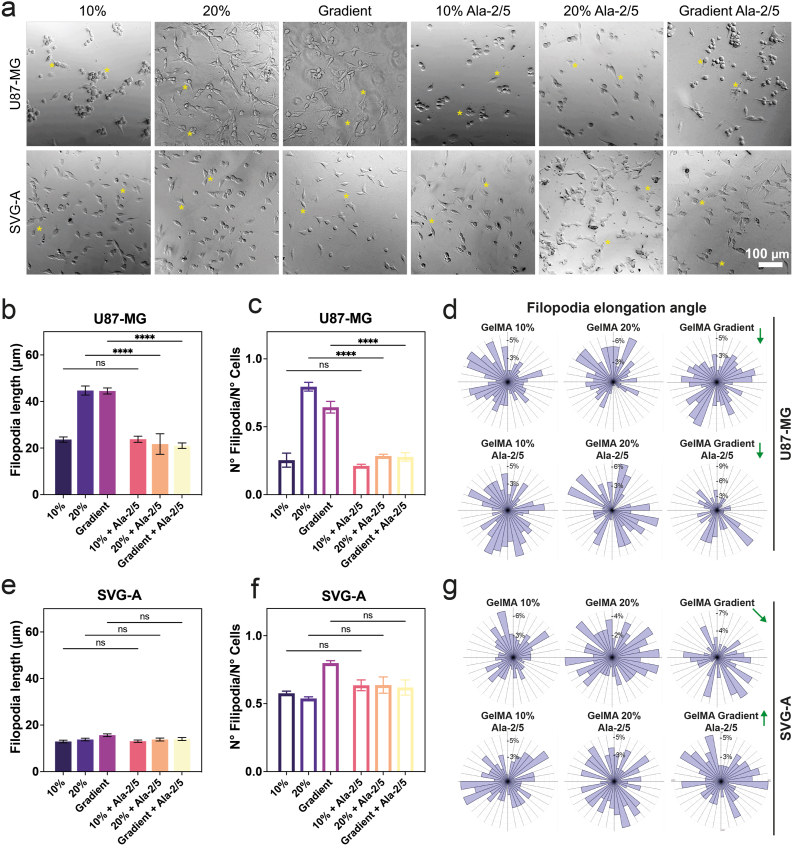
a) Representative confocal micrographs of U87-MG (top) and SVG-A (bottom) cells during 14 h migration on GelMA 10% w/v, 20% w/v, and stiffness-gradient substrates, with or without Ala-2/5 treatment, highlighting differences in filopodia morphology and abundance. Scale bar 100 μm. Yellow asterisks indicate some of the filopodia structures. Quantification of filopodia length (b,e), number of filipodia per cell (c,f), and filopodia elongation angle (d,g) for U87-MG (top) and SVG-A (bottom) cells. Filopodia elongation angles are displayed as rose plots. In gradient substrates, the direction from softer to stiffer regions is indicated by a green arrow. Over 300 filopodia were analyzed per sample. n = 3, the analysis was obtained from at least three independent experiments. Values denote mean ± SEM. ∗p < 0.05; ∗∗p < 0.01; ∗∗∗p < 0.001; ∗∗∗∗p < 0.0001. ns = not significant.

To further investigate the mechanisms underlying cell migration, we analyzed filopodia, the actin-rich protrusions that guide directional movement [37,38], of U87-MG and SVG-A cells cultured on GelMA substrates treated or not with the Ala-2/5 peptide. Representative micrographs of the analyzed regions are shown in Fig. 9a. The analysis revealed that Ala-2/5 differentially affects filopodia length and number in U87-MG cells depending on substrate stiffness. In untreated conditions, cells develop filopodia with lengths of 23.7 ± 1.1 μm, 44.7 ± 1.9 μm, and 44.5 ± 1.3 μm on 10%, 20%, and gradient GelMA substrates, respectively. While no significant change in filopodia length is observed on softer substrates upon treatment (23.8 ± 1.3 μm), a marked decrease is detected on stiffer and gradient substrates (21.7 ± 4.4 μm and 21.1 ± 1.2 μm, respectively). Similarly, Ala-2/5 affects the average number of filopodia per cell. Untreated cells exhibited values of 0.25 ± 0.05, 0.79 ± 0.03, and 0.64 ± 0.04, whereas treated cells showed reduced values of 0.21 ± 0.01, 0.28 ± 0.01, and 0.28 ± 0.03 on 10%, 20%, and gradient substrates, respectively. Thus, consistent with the length measurements, Ala-2/5 does not significantly affect filopodia number on 10% GelMA, but it markedly reduces it on 20% and gradient substrates. Filopodia formation does not display a preferred orientation on substrates with uniform stiffness, whereas a slight tendency toward alignment along the gradient is observed (Fig. 9d–g). In contrast, Ala-2/5 does not significantly affect filopodia length or number in SVG-A cells, which exhibit an average length of ∼14 μm and ∼0.6 filopodia per cell. As observed for U87-MG cells, a mild preferential orientation along the gradient direction is also evident for SVG-A cells.

## 4 Discussion

GBM is a highly malignant brain tumor with a median survival of only 15 months, largely due to its aggressive infiltration into surrounding brain tissue. Tumor cell migration is driven by three coordinated processes: adhesion to and modification of the extracellular matrix (ECM), cytoskeletal reorganization for motility, and enzymatic degradation of matrix proteins to enable invasion. Multiple mechanisms contribute to GBM spread, with cell motility being the key factor underlying tumor cell migration [39,40]. Therefore, the development of new therapies to block tumor cell migration is crucial and could represent a significant step forward in the treatment of GBM. To date, no anti-migratory therapies have been approved for clinical use in GBM patients [41]. However, this therapeutic strategy is attracting increasing attention within the scientific community. A growing number of publications and preclinical studies are investigating both novel compounds and repurposed drugs, originally developed for other diseases, for their ability to limit GBM cell migration [42]. It is within this context that the development of standardized methods for in vitro testing of anti-migratory molecules is essential to make high throughput, faster, and more reliable drug screenings. Such approaches would help reduce the costs of developing new therapies, accelerate the translation from bench to bedside, and minimize the reliance on animal models.

Several platforms and methods for studying cell migration have been previously developed, including 2D cell culture on plastic substrates (e.g., scratch test or Boyden chamber) [20,43], microfluidic lab-on-chip [35,44], polymeric micro- and nanofibers [45,46], as well as 2D and 3D hydrogel substrates [36,47,48]. While all these approaches provide reliable results and valuable strategies to investigate cell migration, they present practical limitations when applied to drug testing. Conventional 2D culture on plastic substrates fails to reproduce key features of the tumor niche, as cells grow on materials that are several orders of magnitude stiffer than brain tissue. Lab-on-chip devices, fibrous substrates more closely mimic aspects of the tumor extracellular matrix, but their fabrication requires complex, expensive setups and highly specialized personnel [35,44].

In contrast, the system proposed in this work relies on simple, minimal and easily reproducible technologies that can be implemented in any laboratory; indeed, it does not require supporting structures and relies on readily available materials. Simplicity does not compromise versatility, as the platform is fully compatible with standard cell culture workflows and supports all major imaging modalities. Substrate fabrication for cell culture is rapid and adaptable to the preparation of multiple samples, enabling high-throughput screening of several drugs in parallel.

The use of GelMA provides additional advantages. It can be synthesized in-house from gelatin through a simple and reproducible process [24]. Its structure ensures the presence of Arg-Gly-Asp (RGD) amino acid sequences within gelatin chains, conferring superior biological performance compared to synthetic polymers [49]. As a result, the substrates inherently provide adhesion sites for GBM cells without the need for post-processing treatments or ECM protein functionalization, even though the matrix can still be enriched with specific proteins to enhance tumor niche biomimicry. Moreover, GelMA stiffness falls in the range of GBM tumor stiffness (from 13.5 to 45 kPa) [13,14] and it can be easily tuned by adjusting the biopolymer concentration, thereby tailoring the platform to specific tests or to other tumor cells originating from different tissues. Finally, the obtained substrates are highly transparent, allowing straightforward optical and fluorescence microscopy analyses. Here we characterized the mechanical properties of the substrates employing two different strategies: (i) compressive tests, that provide a macroscopic evaluation of hydrogel stiffness, and (ii) force/distance AFM measurements, that assess the microscopic mechanical properties of the substrate surface. The first analysis revealed a general trend of increasing stiffness as the hydrogel concentration rises from 10% to 20% w/v. Substrates at 5% w/v were the only exception to this pattern. This is likely related to the supramolecular arrangement of the GelMA chains in the presence of saline solutions, like PBS or culture medium. Indeed, previous studies [30] have shown that interactions between the carboxylic groups of gelatin and cations from the soaking solution reduce the number of free groups available for hydrogen bonding with surrounding water molecules [31], leading to shrinkage and limited swelling and, consequently, to an increase in bulk stiffness.

Interestingly, this behavior was not observed in the force/distance measurements, which instead showed a clear concentration-related increase of Young's modulus from 5% to 20% w/v GelMA. In addition, the modulus values obtained from these measurements were about an order of magnitude lower than those from compressive tests. This discrepancy arises from fundamental differences in testing scale, the localized nature of the deformation, and the influence of microstructural features that bulk mechanical tests cannot capture. Microcracks, micro and nano-pores, or heterogeneity in the material can indeed influence the results of tip-induced deformation more significantly than in bulk compression [50]. In our view, the AFM-based stiffness measurements reported here closely reflect how cells perceive their mechanical environment. The dimensions of the AFM cantilever tip were comparable to those of focal adhesion complexes, effectively mimicking the mechano-sensing processes that cells use to probe their surroundings. Consistent with this, when examining how GelMA stiffness influences the morphology of U87-MG and SVG-A cells, we observed that 5% GelMA substrates induce a predominantly rounded morphology in both cell types, indicating limited spreading. But, as substrate stiffness increases, cells exhibited progressively greater spreading and adopted a more elongated shape. This behavior is consistent with the stiffness variations detected by AFM.

Based on these findings, the 5% sample was excluded from further analysis because its low stiffness failed to support adequate cell adhesion. In contrast, the 10% and 20% substrates fall within a functional stiffness range in which both cell adhesion and substrate–cell interactions are adequately maintained, although at different intensities. Therefore, these two concentrations were selected for the migration assays to evaluate how different stiffness levels influence cell movement.

These tests demonstrated that U87-MG migration speed is of about 0.17 μm/min and 0.2 μm/min on 10% and 20% w/v substrates, respectively. Previous studies on GBM cells reported a wide range of measured speed, from 0.025 to 0.6 μm/min, depending on substrate stiffness, surface functionalization, and composition [35,39,51,52]. Interestingly, when patient-derived GBM cells were studied within a 3D collagen gel matrix, the observed average invasion speed ranged between 0.12 and 0.39 μm/min [53], values comparable to those obtained on our GelMA substrates. Similar findings were reported for fluorescently labeled glioma cells implanted in the rat cerebral cortex and analyzed by time-lapse in vivo microscopy: motility measurements revealed a migration speed of approximately 0.2 μm/min [54]. These results closely match with those measured in our study and indicate that our substrates successfully reproduce physiologically relevant mechanical properties of brain tissue and provide reliable and representative information on cell motility.

Moreover, we observed that glioblastoma cells display higher migration speeds than non-tumoral astrocytes, reflecting their well-known locally invasive phenotype and increased motile capacity [55,56], which is consistent with the highly infiltrative nature of glioblastoma in the brain.

We also compared the motility of untreated cells with that of cells exposed to the Ala 2/5 cyclopeptide [20,21]. This is a peptide derived from a naturally occurring sequence of human urokinase, binding with high affinity and specificity to αV-integrins and inhibiting cell migration in organotypic systems and in vivo [23]. This clearcut inhibitory effect is reproducible also on 10% and 20% GelMA substrates, where Ala 2/5 effectively reduced U87-MG migration velocity and accumulated distance, with the strongest effect observed on 10% GelMA. Notably, on 10% GelMA substrates this peptide showed no detectable effect on SVG-A cells, suggesting its potential selectivity toward malignant GBM cells, while limiting its action on non-tumoral glial cells counterparts.

Leveraging the simplicity of our fabrication strategy, we further improved the testing platform by developing an easy and reproducible method to generate substrates with a stiffness gradient between 1 and 6.5 kPa, evaluated by AFM force/distance measurements. By adding long-chain fluorescent dextran-FITC to one of the two GelMA solutions, we were able to easily visualize the stiffness gradient through fluorescence microscopy.

Literature reports several examples of stiffness gradient substrates employed in durotactic evaluations, realized by photopolymerization strategies [57,58] employing movable [59,60] or graded photomasks [48,61], soft lithographic or mold based approaches [48,58], microfluidic and lab-on-chip devices [62], 3D printing and bioprinting [63,64] and hybrid approaches [35,65]. However, most of these approaches are relatively complex and require expensive instrumentation and dedicated fabrication facilities. For instance, photopolymerization-, soft lithography-, and lab-on-chip-based methods generally involve preliminary photolithographic fabrication steps requiring mask aligners, specific UV light sources, and dedicated optical setups, often located in clean-room environments. Consequently, these techniques rely on highly specialized personnel and costly fabrication infrastructures, making them overall more complex than the strategy proposed in the present work.

Simpler approaches have also been reported. In particular, Lo and colleagues described a method conceptually similar to ours, based on the compression of two polyacrylamide hydrogel drops using a coverglass [66]. However, the resulting stiffness gradient was highly localized, with a transition region between the low- and high-stiffness areas estimated to be only 50–100 μm wide. Such a narrow interface considerably limits the number of analyzable cells, since only cells located near the boundary can be evaluated.

In contrast, while maintaining a similarly simple fabrication strategy, the use of GelMA in our platform enables the generation of a continuous stiffness gradient extending across the entire substrate surface. This allows the analysis of a substantially larger number of cells and, in our experiments more than 150 cells were analyzed per sample, with at least three samples evaluated for each experimental condition. This provides a statistically robust platform for drug testing, capable of detecting even subtle variations in migratory behavior.

On gradient substrates as well, U87-MG cells exhibit a more pronounced migration speed compared to SVG-A cells, confirming the previously obtained results. However, it is important to emphasize that the two cell types interact with different surfaces, since only SVG-A cells require collagen coating for proper adhesion to the substrate. Nevertheless, force/distance AFM analyses demonstrated that the functionalization does not alter the topographical or mechanical properties of the substrate, thus we can suppose that the collagen layer deposited on the surface has a very limited thickness, sufficient to promote SVG-A cell adhesion, but not sufficient to significantly alter the overall surface stiffness and morphology. Indeed, we demonstrated the capability of both U87-MG and SVG-A cells to sense this stiffness gradient, with SVG-A exhibiting a more pronounced directional migration. This behavior would not be expected if any functionalization inhomogeneities were masking the mechanical properties of the underlying substrate. On the contrary, it could indicate that the presence of collagen enhances cellular sensing of substrate stiffness. Indeed, given that gelatin (and therefore GelMA) is derived from the hydrolytic degradation of collagen, it is reasonable to assume that it retains structural motifs capable of promoting non-covalent interactions with collagen [67]. Considering that collagen is essential for SVG-A cell adhesion, the expression of collagen-specific integrins can be expected on the membrane of these cells. In this context, collagen may act as an interfacial mediator between GelMA stiffness and the cells, thereby enhancing mechano-sensing and promoting durotactic behavior.

The angular distribution of cell movements showed a clear directional migration from softer toward stiffer regions of the substrate, in line with previous findings on mechano-transduction phenomena in solid tumors [13,35,68]. These findings demonstrate that the proposed platform not only supports the study of durotaxis but also enables the assessment of cell-specific responsiveness to substrate stiffness, a key parameter for fully understanding cell-substrate interactions. Motility analysis on the graded substrates also confirmed the inhibitory effect of Ala 2/5 on cell migration, with 45% and 30% reduction in migration velocity for U87-MG and SVG-A cells respectively.

Migration inhibition mechanism related to Ala-2/5 action was further investigated analyzing the filipodia morphometric features. Interestingly, Ala-2/5 reduces filopodia length and number in U87-MG cells with a stiffness-dependent manner, showing minimal effects on soft (10%) substrates but a strong inhibition on stiffer (20%) and gradient GelMA conditions. Conversely, SVG-A cells filopodia are largely insensitive to Ala-2/5. This behavior is likely due to the functional relevance and overexpression in GBM cells of αV-containing integrins, the target of Ala-2/5 [21], which are critically involved in tumor invasion. In particular, integrins such as αVβ3 and αVβ5 are upregulated in GBM compared to normal brain tissue and contribute to tumor progression and enhanced migratory capacity [69].

In contrast, in astrocytes αV integrin expression appears to be less abundant. Generally, astrocytes express a broad repertoire of integrins, including β1-containing receptors that mediate adhesion to extracellular matrix components such as collagen and laminin [[70], [71], [72]]. As a result, the presence of integrin populations not targeted by the peptide, likely allows alternative adhesion pathways to remain active in SVG-A cells even after Ala-2/5 administration. Consequently, inhibition of αV integrins results in a stronger effect in glioblastoma cells, leading to a marked reduction in migration speed and in the formation of cellular protrusions.

It is important to note that the binding of uPAcyclin II to αV subunit does not follow the canonical RGD-binding mode [21]. Therefore, the RGD sequences within the GelMA matrix are not expected to directly compete with the peptide for the binding site. However, we cannot exclude that GelMA-derived RGD signals may indirectly alter the expression or the activation state of αV subunits or downstream mediators (e.g., pp125FAK, p-paxillin), thus modulating SVG-A sensitivity to anti-αV integrin cyclopeptide. Although the mechanism underlying the reduced sensitivity of SVGA cells to the inhibition by the cyclopeptide deserves further investigation, it is likely to be related to the expression level/activation of the target integrin and/or downstream signaling mediators involved in the inhibitory activity.

Overall, these data confirm the Ala-2/5 cyclodecapeptide as a valid lead compound for the development of novel potent anti-GBM agents. To the best of our knowledge, this is the first demonstration of its performance in a mechanically controlled microenvironment. Previous studies [[20], [21], [22]] have assessed this molecule using conventional plastic-based assays, which fail to recapitulate relevant physical features of the tumor microenvironment. The present platform therefore provides a more physiologically relevant testing context and highlights its potential for preclinical screening applications of anti-migratory drugs. By enabling reliable and efficient evaluation of novel compounds, such platforms could accelerate the research and development of new therapeutic strategies against GBM.

## 5 Conclusions

Current therapies against GBM do not adequately target its high migratory behavior and invasion capacity, highlighting the need for effective anti-migratory therapies and reliable in vitro models for their testing. Hence, in this study, we developed a simple, low-cost, and reproducible in vitro platform that mimics essential mechanical features of the GBM niche. By tuning GelMA concentration, we obtained highly flat and transparent substrates with uniform stiffness or a controlled stiffness gradient, suitable for live-cell imaging and quantitative analysis of cell migration. We demonstrated that substrate stiffness significantly influences cell morphology, spreading, and motility and we also validated the possibility to apply these substrates for the study of anti-migratory drugs, testing the cyclopeptide Ala-2/5 on U87-MG glioblastoma cells and SVG-A astrocyte cells. The study revealed a clear anti-migratory effect affecting cell velocity on both cell types, with a greater impact on tumor cells. Overall, this work demonstrates that this hydrogel-based system can bridge the gap between simple 2D assays and complex advanced models, providing a practical and scalable tool for mechanobiology studies and for the preclinical screening of anti-migratory drugs to be used in multitarget therapies against invasive cancers.

## CRediT authorship contribution statement

**Laura Sercia:** Conceptualization, Data curation, Formal analysis, Investigation, Methodology, Validation, Writing – original draft. **Alberto Portone:** Conceptualization, Formal analysis, Investigation, Methodology, Visualization, Writing – original draft. **Stefano Leporatti:** Methodology, Visualization, Writing – review & editing. **Stefania Belli:** Methodology, Writing – review & editing. **Paola Franco:** Conceptualization, Methodology, Writing – review & editing. **Maria Patrizia Stoppelli:** Conceptualization, Funding acquisition, Project administration, Writing – review & editing. **Giuseppe Gigli:** Conceptualization, Funding acquisition. **Alessandro Polini:** Conceptualization, Funding acquisition, Project administration, Supervision, Writing – review & editing. **Francesca Gervaso:** Conceptualization, Funding acquisition, Project administration, Supervision, Writing – review & editing.

## Declaration of competing interest

The authors declare that they have no known competing financial interests or personal relationships that could have appeared to influence the work reported in this paper.

## Data Availability

Data will be made available on request.
