# Supplementary material for: Simple fabrication method for cancer cell migration studies on biomimetic substrates with tunable stiffness
Source: Mater Today Bio. 2026 Jun 8;39:103332. doi: 10.1016/j.mtbio.2026.103332 (PMC13267613; doi:10.1016/j.mtbio.2026.103332)
Supplement: Multimedia component 1 [file mmc1.docx]

**Supplementary data**

**Simple Fabrication Method for Cancer Cell Migration Studies on Biomimetic Substrates with Tunable Stiffness**

Laura Sercia,^1,2,3,§^ Alberto Portone,^1,3,§^ Stefano Leporatti,^1,3^ Stefania Belli,^4^ Paola Franco,^4^ Maria Patrizia Stoppelli,^4,5^ Giuseppe Gigli,^1,3,6^ Alessandro Polini,^1,3,†,*^ and Francesca Gervaso^1,3,†,*^

1. Institute of Nanotechnology, National Research Council (CNR-NANOTEC), c/o Campus Ecotekne, via Monteroni, 73100 Lecce, Italy
2. University of Salento, Dipartimento di Ingegneria dell'Innovazione, c/o Campus Ecotekne, via Monteroni, 73100 Lecce, Italy
3. Tecnomed Puglia – Technopole for Precision Medicine (Biotech Lecce Hub), c/o Ecotekne Campus, via Monteroni, 73100 Lecce, Italy
4. Institute of Genetics and Biophysics “A. Buzzati Traverso” (CNR-IGB), National Research Council, 80131 Naples, Italy
5. UniCamillus-Saint Camillus International University of Health Sciences, Departmental Faculty of Medicine and Surgery, 00131 Rome, Italy
6. University of Salento, Dipartimento di Medicina Sperimentale, c/o Campus Ecotekne, Via Monteroni, 73100 Lecce, Italy

§ These authors contributed equally to this work as first authors.

^†^ These authors contributed equally to this work as senior authors.

^*^ Corresponding authors: alessandro.polini@cnr.it (AP), francesca.gervaso@cnr.it (FG).

[alessandro.polini@cnr.it](mailto:alessandro.polini@cnr.it)

[francesca.gervaso@cnr.it](mailto:francesca.gervaso@cnr.it)

Keywords: cell migration assay, in vitro models, glioblastoma, anti-migratory drugs, hydrogel stiffness, mechanobiology.


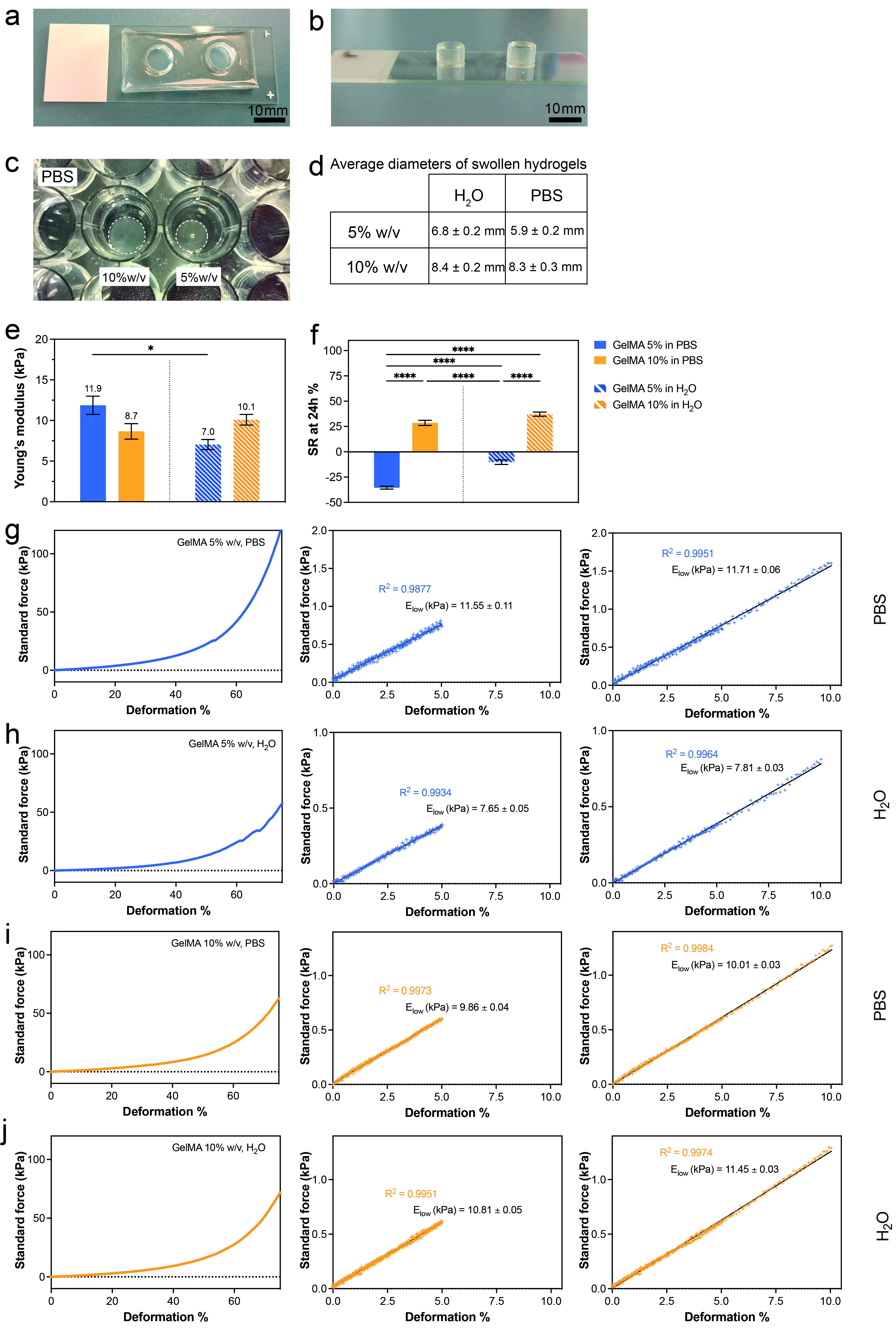


**Figure S1.** Swelling and compressive behavior of GelMA 5 and 10% w/v hydrogels after 24 h incubation in either H_2_O or PBS. a,b) Picture of the PDMS mold (a) used to obtain the hydrogel samples (b) after UV crosslinking. c) Picture of GelMA 5% and 10% w/v hydrogels after swelling in PBS highlighting the difference in sample size. d) Average diameters of swollen GelMA 5% and 10% w/v hydrogels in either dH_2_O or PBS for 24 h at 37°C, n = 6. e) Young’s Modulus of GelMA 5% and 10% w/v hydrogels after 24 h incubation in either PBS or dH_2_O, n = 6. f) Swelling ratio (SR) percentage of GelMA 5% and 10% w/v hydrogels after 24 h incubation in either PBS or dH_2_O, n = 6. Values denote mean ± SEM. * p<0.05; ** p<0.01; *** p<0.001, **** p<0.0001; no bar = not significant.


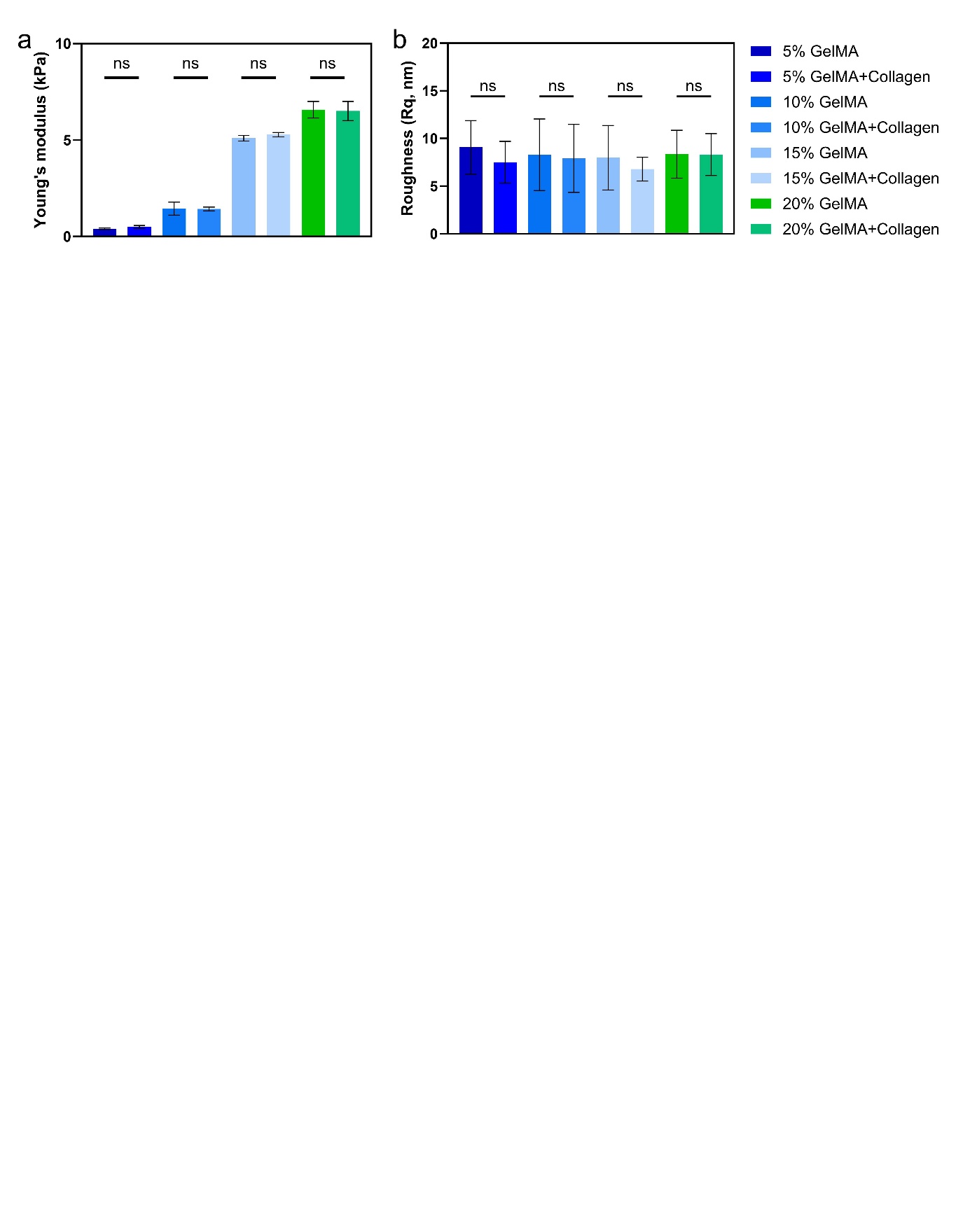


**Figure S2.** Comparison of stiffness (a) and roughness (b) in substrates with and without collagen functionalization. Stiffness values were collected through force/distance measurements in QI mode AFM (5×10 µm^2^, 30×60 pixels), with every pixel corresponding to a force/distance measurement. g) Topographic surface roughness (Rq, root-mean-square) of the substrates were evaluated by tapping mode AFM (5×10 µm^2^). At least three areas for every sample type were analyzed. Results are reported as mean ± SEM. ns = not significant.


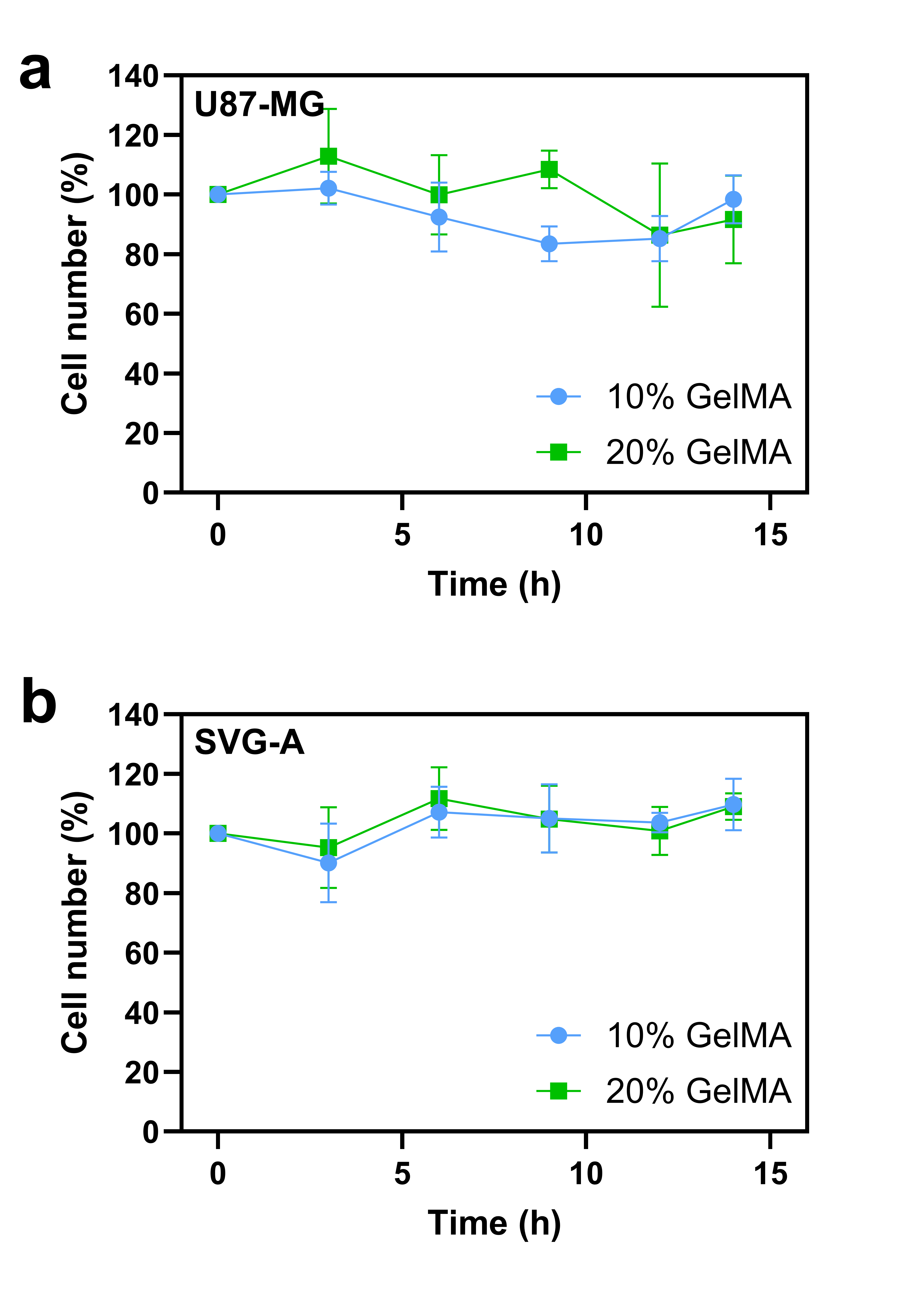


**Figure S3.** U87-MG (a) and SVG-A (b) number of cells were measured in three independent brightfield micrographs at different time points of the migration analysis (0, 3, 6, 9, 12 and 14 hours) on 10% and 20% GelMA substrates. Cell numbers were normalized to the initial value (t = 0, set to 100%). No evident growth trends were observed, indicating that the analysis time was insufficient to detect proliferation-related effects. Values denote mean ± SD.


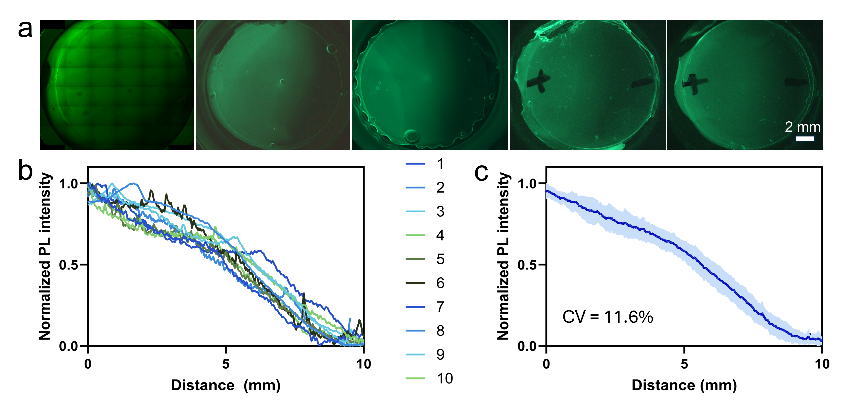


**Figure S4.** Assessment of the reproducibility of the proposed fabrication process. Fluorescence images from ten distinct substrates, fabricated at different times for separate experiments, were acquired using varying microscopy systems and imaging parameters. Five representative examples are shown (a). From left to right, the first sample was acquired using automated tile scanning on a confocal microscope, while the remaining four were imaged with a fluorescence stereo zoom microscope. (b) Comparison of substrates photoluminescence (PL) intensity profiles along 10 mm lines spanning from the stiffer to the softer regions, highlighting the gradient trend. Although the images were acquired using different microscopes and settings (e.g., exposure time and excitation intensity), the profiles can be directly compared by normalizing the intensity signal between 0 and 1. This procedure minimizes the influence of non-specific background fluorescence and acquisition parameters, highlighting the gradient trend. For each position x, we calculated the PL intensity mean and standard deviation across the ten profiles; the corresponding plot is shown in (c). The coefficient of variation (CV = SD/mean) is reported in the graph, indicating good reproducibility of the fabrication process.


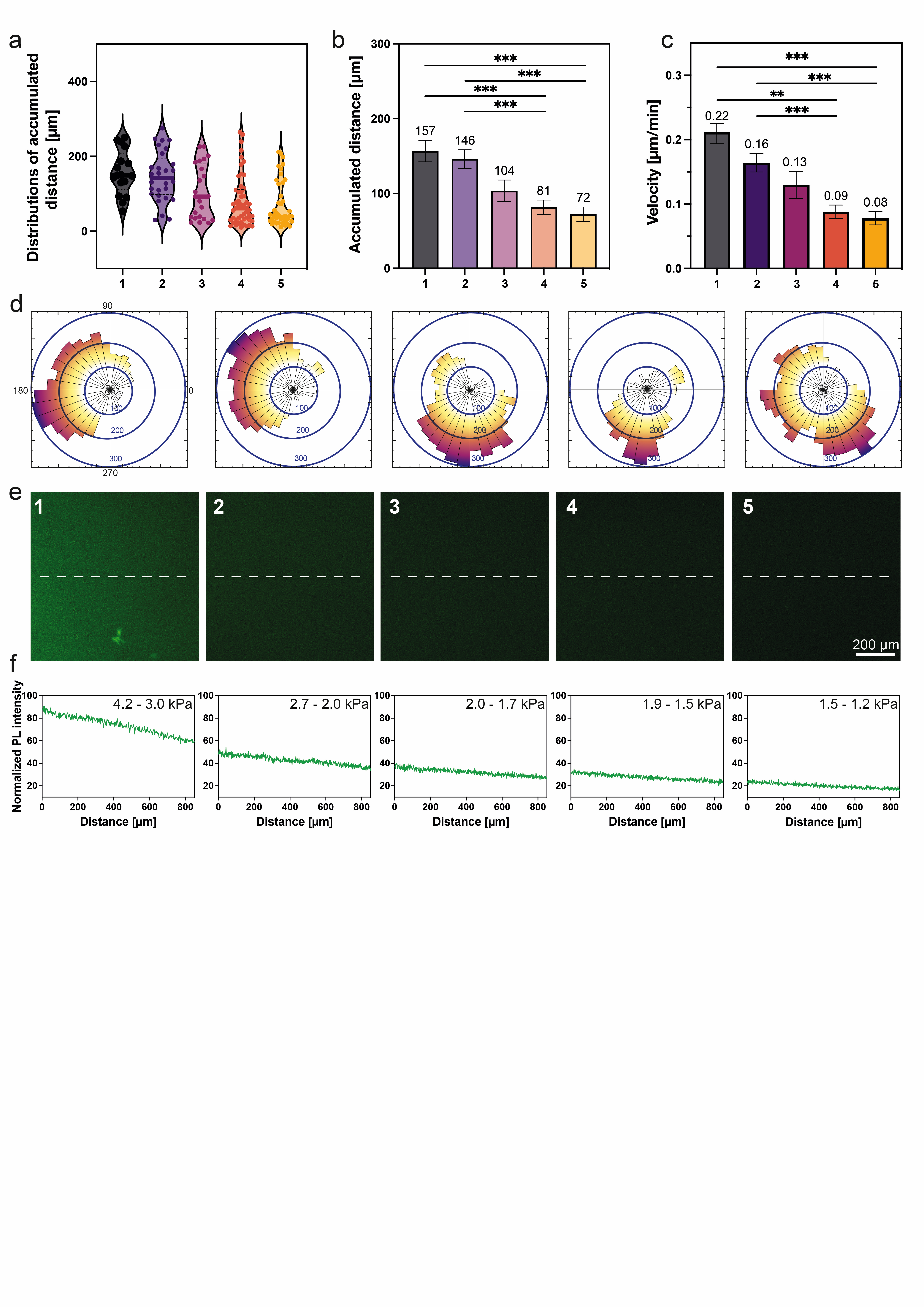


**Figure S5.** Quantitative analysis of U87-MG cell migration along a GelMA stiffness-gradient substrate. a-c) Histograms reporting the distribution of accumulated distance (a), average accumulated distance (b), and migration velocity (e) in the five analyzed stiffness-gradient areas (e) of the same samples. (n =50, number of analyzed cells per area). Values denote mean ± SEM. * p<0.05; ** p<0.01; *** p<0.001. (d) Rose plots showing the angular distribution of all movements within the analyzed tracks. Blue circles indicate the frequency of movements associated with each angle. (e) Fluorescent images of the stiffness-gradient areas (1-5) used for the cell migration analysis. The green signal is due to the presence of dextran-FITC in GelMA 20% solution and marks the stiffness gradient that grows with PL intensity. (f) Normalized PL intensity maps along the dashed lines (e) of the stiffness-gradient areas 1-5. The insets in (f) show the stiffness range in the relative area, calculated using the formula reported in the Material section (E = 4.1 PL_0-1_+ 0.554) which correlates the normalized PL intensity and Young’s modulus.
